# Climate change and anthropogenic food manipulation interact in shifting the distribution of a large herbivore at its altitudinal range limit

**DOI:** 10.1038/s41598-021-86720-2

**Published:** 2021-04-07

**Authors:** Julius G. Bright Ross, Wibke Peters, Federico Ossi, Paul R. Moorcroft, Emanuele Cordano, Emanuele Eccel, Filippo Bianchini, Maurizio Ramanzin, Francesca Cagnacci

**Affiliations:** 1 Department of Organismic and Evolutionary Biology, Harvard University, Cambridge, MA USA; 2 Wildlife Conservation Research Unit, Department of Zoology, University of Oxford, Oxford, UK; 3 Department of Biodiversity, Conservation and Wildlife Management, Bavarian State Institute of Forestry, Freising, Germany; 4 Department of Biodiversity and Molecular Ecology, Research and Innovation Centre, Fondazione Edmund Mach, San Michele all’Adige, Italy; 5 C3A - Centro Agricoltura Alimenti Ambiente, Università degli Studi di Trento, San Michele all’Adige, Italy; 6 Rendena100, Engineering and Consultancy sole proprietorship, Tione di Trento, Italy; 7 Department of Sustainable Agro-Ecosystems and Bioresources, Research and Innovation Centre, Fondazione Edmund Mach, San Michele all’Adige, Italy; 8 Department of Biology and Biotechnology ‘Charles Darwin’, University of Rome ‘La Sapienza’, Rome, Italy; 9 Department of Agronomy, Food, Natural resources, Animals and Environment, University of Padova, Padova, Italy; 10 Present Address: Wildlife Conservation Research Unit, Department of Zoology, University of Oxford, Oxford, UK

**Keywords:** Behavioural ecology, Climate-change ecology, Animal behaviour, Ecology, Zoology, Ecology, Behavioural ecology, Climate-change ecology, Climate sciences, Climate change, Climate-change impacts

## Abstract

Ungulates in alpine ecosystems are constrained by winter harshness through resource limitation and direct mortality from weather extremes. However, little empirical evidence has definitively established how current climate change and other anthropogenic modifications of resource availability affect ungulate winter distribution, especially at their range limits. Here, we used a combination of historical (1997–2002) and contemporary (2012–2015) Eurasian roe deer (*Capreolus capreolus*) relocation datasets that span changes in snowpack characteristics and two levels of supplemental feeding to compare and forecast probability of space use at the species’ altitudinal range limit. Scarcer snow cover in the contemporary period interacted with the augmented feeding site distribution to increase the elevation of winter range limits, and we predict this trend will continue under climate change. Moreover, roe deer have shifted from historically using feeding sites primarily under deep snow conditions to contemporarily using them under a wider range of snow conditions as their availability has increased. Combined with scarcer snow cover during December, January, and April, this trend has reduced inter-annual variability in space use patterns in these months. These spatial responses to climate- and artificial resource-provisioning shifts evidence the importance of these changing factors in shaping large herbivore spatial distribution and, consequently, ecosystem dynamics.

## Introduction

In temperate and boreal ecosystems, both the latitudinal and altitudinal distribution of species are often determined by winter conditions^1,2^. Ungulates and other large herbivores are particularly challenged by the presence of snow, which decreases food accessibility^3^, affects vegetation phenology^4^, increases the costs of thermoregulation^5^, and hampers movement^6^. While these constraints have driven some morphological adaptation in ungulates living in snow-covered environments^7^, most temperate ungulates rely on space-use plasticity to meet the challenges posed by snow. Winter space-use tactics, whether migration^8^ or refuges within winter ranges^9^, minimize the costs associated with winter conditions and are therefore crucial to ungulate survival in temperate regions.

Ongoing changes in winter conditions stemming from anthropogenic climate change thus carry extensive implications for space use in ungulates^10^, and are most evident at the latitudinal and altitudinal range limits of species^11^. These changes, and particularly a reduction in the snowpack^12^, have been particularly noticeable in the mid-elevations (~ 1000–1700 m, here and hereafter given as above sea level) of mountainous regions in the Northern Hemisphere such as the European Alps^12^, which form the altitudinal range limit of many species. Moreover, in these alpine regions, climatic changes often co-occur with the alteration of ungulate resource availability through supplemental feeding^13,14^. This practice consists of the provisioning of abundant plant-based food (grain or maize seeds, grain and fiber pellets, hay, fruit), usually placed in ad hoc feeding sites during winter months. Thus, the natural distribution of food resources, normally scarce in winter, is altered by point-source supplies of highly concentrated food^15^. In spite of its high ecological relevance, studying the interplay between long-term anthropogenic changes in the physical environment (the snowpack) and food availability (supplemental feeding) in shaping ungulate space use patterns is challenging, due to the gradual nature of these changes and the resulting need to have comparable measurements of space use that have been collected many years apart. Such datasets are rare, as the advent of large herbivore tracking technology is relatively recent^16^.

**Figure 1:**
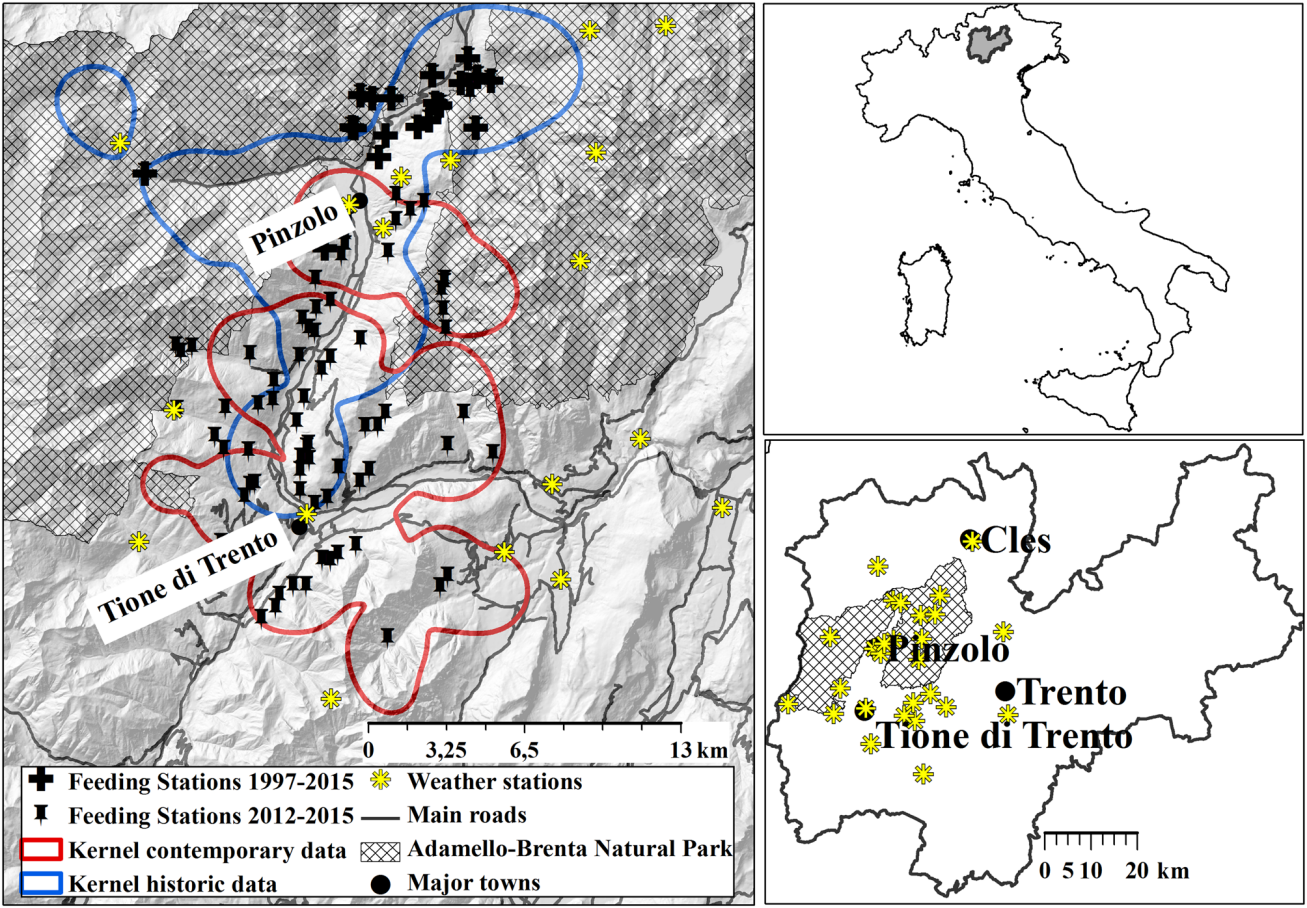
Study area in the Italian Alps, with insets of Italy (top right) and Trentino Province (bottom right). Polygons are shown for the overlapping historical (blue) and contemporary (red) roe deer population (kernel densities 99% based on radio-telemetry relocations). Supplemental feeding sites are indicated for each period as well as the 23 weather stations used to run GEOtop simulations (Supplementary S2).

**Figure 2:**
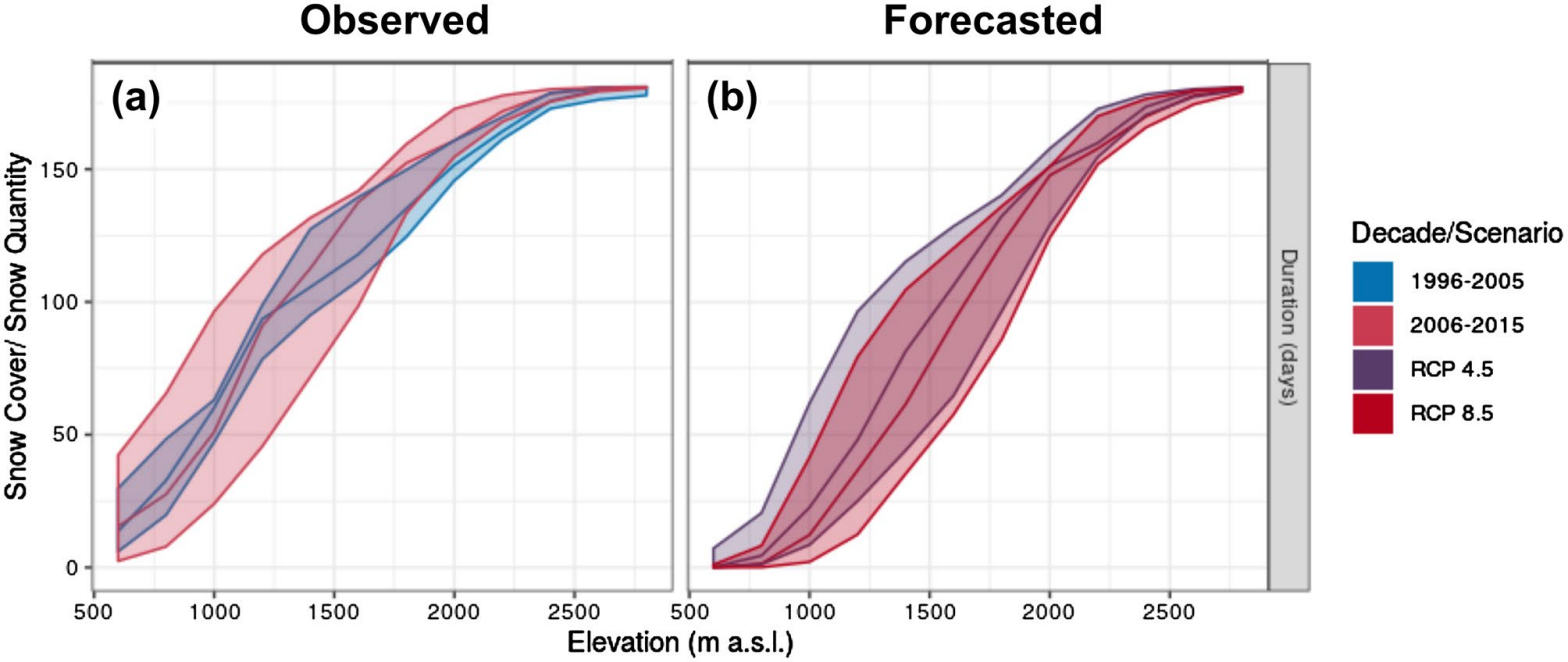
Snow cover duration for each winter season (Nov–Apr) as a function of altitude in the study area, calculated using GEOtop 2.0 (Supplementary S2). Central continuous lines denote the 50th percentile, while range between 25 and 75th percentiles is shown by the colored areas. RCP scenarios refer to the last decade of the projection period (2046–2065).

In this study, we had the unique opportunity to contrast and compare “historical” (1999–2002) and “contemporary” (2012–2015) space use patterns of Eurasian roe deer (*Capreolus capreolus*) in a valley at the species’ altitudinal range limit in the Italian Alps (Fig. 1) under ongoing and forecasted changes in winter conditions and food availability (Fig. 2). Roe deer exhibit high ecological plasticity^17,18^, making them uniquely suited as a bellwether of the ongoing effects of such changes on ungulate winter spatial distribution. Winter harshness (particularly deep snow) severely restricts roe deer movement and thus its spatial distribution, because of their small body mass (18–49 kg) and short legs (50–60 cm)^6^. Under these constraints, roe deer living in snowy areas adopt movement tactics at multiple spatiotemporal scales. First, roe deer exhibit a seasonal partial migration strategy, with all individuals overwintering in ranges characterized by less extreme snow conditions, and only some migrating to summer ranges (typically at higher elevations)^8^. Second, within winter ranges, we have previously documented third-order selection^19^ for shallow snow depth and dense forest canopy^20,21^. Where supplemental feeding is practiced, the presence of these anthropogenic resources also interacts with winter severity to affect roe deer space use^14^.

In our study area, winter temperatures (Fig. S1.2a) and the variability of snow cover at low-to-intermediate elevations have increased over the last two decades (Fig. 2a), as has the prevalence and distribution of supplemental feeding sites (Fig. 1). We performed an analysis of roe deer space-use data covering this timespan. Specifically, our analysis used (i) a GPS dataset of contemporary roe deer relocations (2012–2015, hereafter “contemporary period”) in a valley in the Italian Alps (Fig. 1); (ii) a VHF dataset of historical roe deer relocations (1999–2002, hereafter “historical period”) in a largely overlapping area^22^; (iii) a detailed mapping of supplemental feeding site distribution and canopy cover in both periods; and (iv) an advanced surface-process predictive hydrological model of snow depth^23^, to fit resource selection functions (RSFs) modelling relative probability-of-use across the study area. We used these RSFs to predict and compare (by means of kappa coefficients^24^ and subtraction maps) the winter (November to April) distribution of roe deer at a high temporal resolution scale (monthly) over the course of the two study periods. Utilizing two climate forecasts of “intermediate” or “severe” thermal loading (the IPCC’s “Representative Concentration Pathways” for 2046–2065, modelling either intermediate or no restraint in emissions; hereafter RCP4.5^25^ and RCP8.5^26^, respectively), we then used the model built with contemporary data to evaluate roe deer habitat selection under predicted future snow depth scenarios. We thus evaluated the following set of hypotheses.

First, we hypothesized that winter distribution would be influenced by both (i) snow depth and (ii) the proximity of supplemental feeding sites^20^. We further hypothesized (iii) that there would be an interaction between these two factors, with the availability of supplemental feeding affecting the response of roe deer to snow depth. Specifically, we expected that the relative probability of space use would decrease with deep snow (> ~50 cm; Prediction 1) and increase in the proximity of supplemental feeding sites (Prediction 2). Following hypothesis (iii), we expected that as the availability of supplemental feeding sites increased between study periods, the relative probability of space use would concentrate around these sites irrespective of the presence of snow (Prediction 3)—i.e., the attraction of supplemental feeding sites at higher frequency would prevail over the negative effects of snow. Consequently, we also expected that the predicted spatial distribution of roe deer would display higher variability between the historical and the contemporary period than within either period, in response to the inter-decadal changes in snow depth and the substantial increase in supplemental feeding site availability across the study area (Prediction 4). Further, because the year-to-year differences within a given period should depend on snow depth variability (given supplemental feeding sites are constant), we predicted that the increases in winter variability in alpine regions (Fig. 2a) would produce more variability within the contemporary period than in the historical one (Prediction 5a), and even further variability under forecasted warming scenarios (Prediction 5b).

## Results

### Snow trends

We observed inter-decadal trends in snow cover duration (days with snow depth > 5 cm) and absolute snow depth, both as a function of elevation and of the winter months (November–April). The decade-aggregated average of “snow cover days” from November–April (Fig. 2) showed higher variability in snow cover duration within our study area from 2006 to 2015 (encompassing the contemporary study period) than in the previous decade (from 1996 to 2005, encompassing the historical study period). This pattern was particularly evident at low-to-intermediate elevations (500–1500 m; Fig. 2a). The same aggregated average also predicts that snow cover duration will decrease as a function of continued climate change, again especially at lower elevations (Fig. 2b). However, the comparison of snow depth between historical and contemporary study periods as a function of elevation (Fig. S2.2) showed that in early (November–December) and late (April) winter, snow depth variability decreased in the contemporary period, as a consequence of scarcer snowfall in these months in recent years. In contrast, the variability of snow depth over the central winter months (January–March) was relatively high during both study periods.

### Roe deer space use patterns

**Figure 3:**
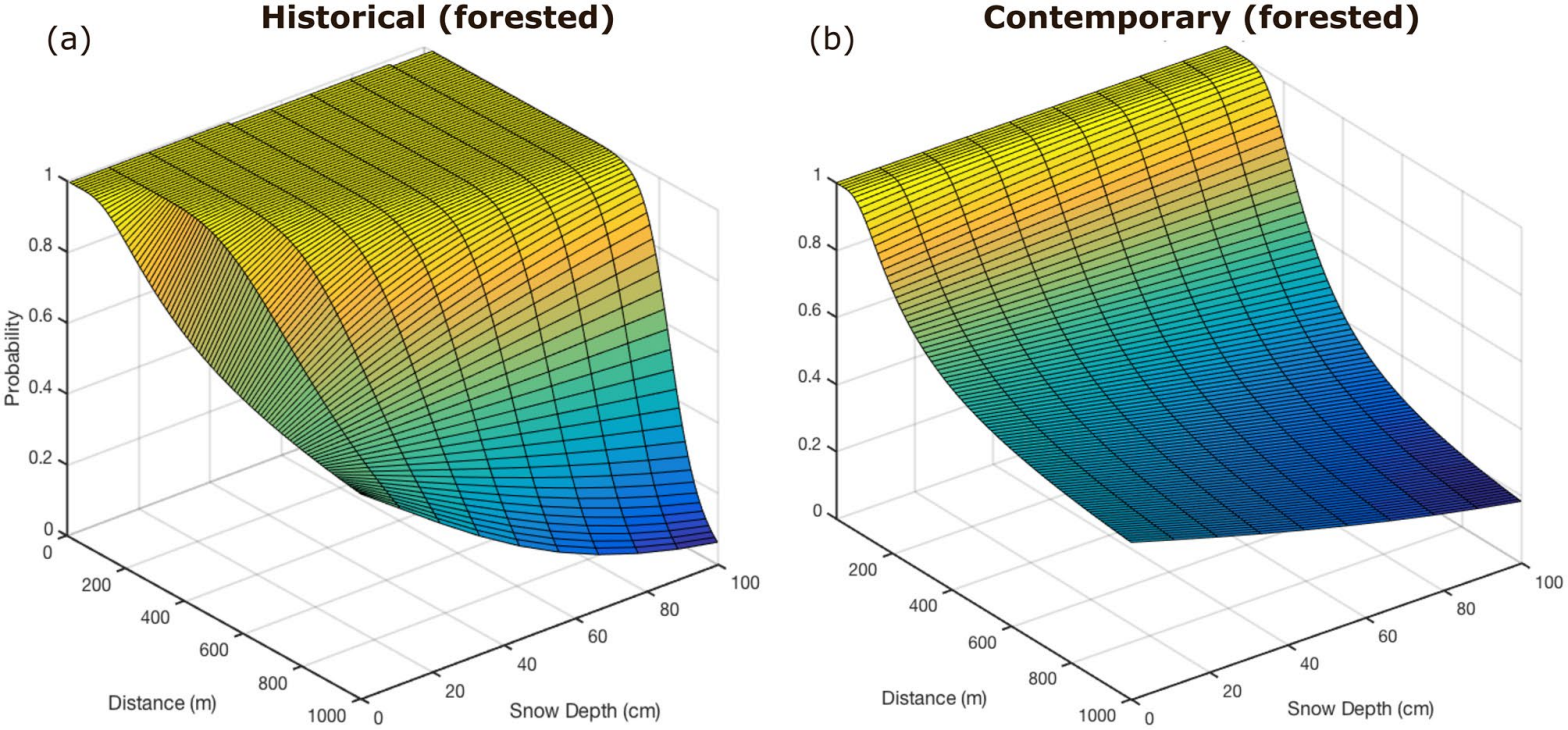
3D prediction plots of relative probability-of-use of forested habitats (for unforested habitats, see Fig. S3.1) in the two periods (1999–2002: (**a**); 2012–2015: (**b**)) covered by the analysis.

Contemporary and historical resource selection functions (hereafter “RSF_c_” and “RSF_h_”, respectively) confirmed the hypothesized effects of snow and supplemental feeding (hypotheses i and ii) on relative probability of space use by roe deer. In particular, roe deer avoided areas with high snow depth (Prediction 1: β_c_ = − 0.018 ± 0.002/cm; β_h_ = − 0.174 ± 0.010/cm, both *p* < 0.001) and selected for areas close to supplemental feeding sites (Prediction 2: β_c_ = 351.750 ± 18.540; β_h_ = 529.691 ± 26.650, both *p* < 0.001, for the reciprocal of distance from the closest feeding site, “*1/DistFS*”). Further, in accordance with hypothesis (iii), the selection for supplemental feeding sites was stronger at higher snow depths, but less so as the availability of supplemental feeding sites increased between periods, as shown by the different magnitude of the interaction terms (Prediction 3: β_c_ = 4.282 ± 0.752; β_h_ = 140.411 ± 8.432, both *p* < 0.001) (Fig. 3 and Fig. S3.1). The models also indicated strong positive selection for canopy cover (β_c_ = 0.8033 ± 0.067; β_h_ = 1.217 ± 0.084, where 0 = open, 1 = forested, reference category: open; both *p* < 0.001), which changed relatively little between study periods.

The robustness of both RSFs for predicting relative probability-of-use under a variety of conditions was established via spatial validation (each RSF in its own study period) and temporal validation (RSF_c_ hindcasted to the historical period, see “Methods” section). Each validation was performed through a Kendall’s tau (*τ*) rank correlation analysis (spatial RSF_h_: *τ* = 0.80, SD = 0.01, *p* < 0.05 for all months; spatial RSF_c_: *τ* = 0.70, SD = 0.23, all *p* < 0.05 except November 2014, *p* = 1; temporal RSF_c_: *τ* = 0.76, SD = 0.13; all *p* < 0.05 except April 1999, *p* = 0.13; see “Methods” section for explanation of *τ* and Supplement S5 for further information). The goodness-of-fit of the fitted RSFs was confirmed by Receiver Operating Characteristic (ROC) area-under-the-curve (AUC) values (AUC_h_ = 0.86, AUC_c_ = 0.82, ROC plots Fig. S3.2) and k-fold cross-validation, also using Kendall’s tau (fivefold, mean historical *τ*_h_ = 0.75, two historical k-fold subsets *p* < 0.001, three subsets *p* < 0.05; mean contemporary *τ*_c_ = 0.96, all contemporary k-fold subsets *p* < 0.001).

### Temporal variation in patterns of space use

**Figure 4:**
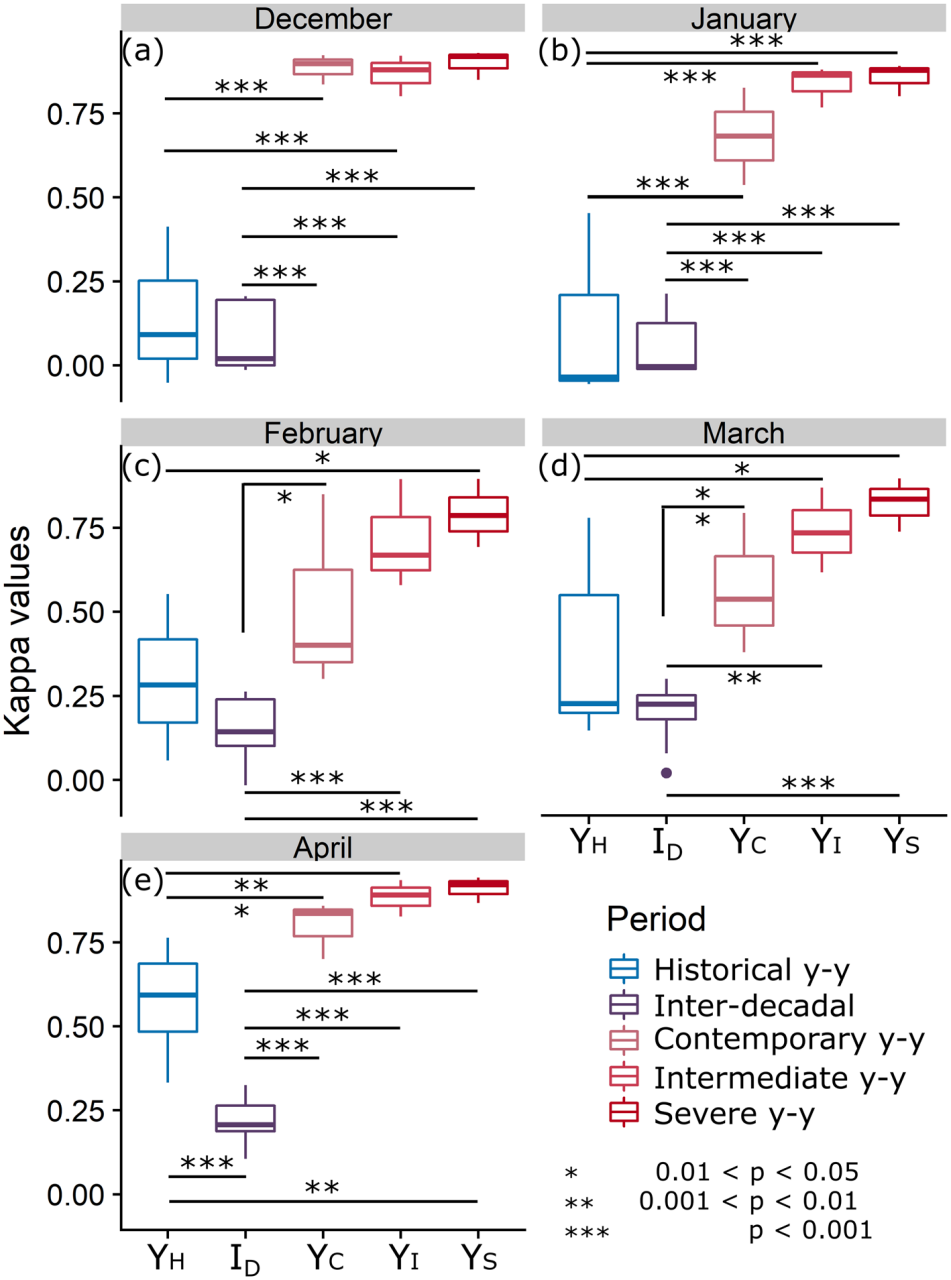
Kappa coefficients comparing the relative probability-of-use by month. Y_H_: year-to-year (y–y in the legend) comparisons for historical study period using RSF_h_; I_D_: inter-decadal comparisons; Y_C_: year-to-year comparisons for contemporary study period using RSF_c_; Y_I_: year-to-year comparisons for intermediate forecast scenario; Y_S_: year-to-year comparisons for severe forecast scenario. Sample size varies based on the number of years for which data was collected in that month (November not shown due to low sample size). Asterisks denote pairwise significance of difference between categories of comparison (***, *p* < 0.001; **, *p* < 0.01; *, *p* < 0.05).

We used kappa coefficients (*κ*) to compare similarity between predicted relative probability-of-use (*Rp*) maps within and between periods (*κ* = 1 for identical maps, *κ* = − 1 for reverse-image maps). These coefficients indicated substantial inter-decadal shifts in *Rp* by roe deer across our study area. In agreement with Prediction 4, inter-decadal variability in *Rp* was significantly higher (lower *κ*) than year-to-year variability in all months of the contemporary period and in April of the historical period (Fig. 4; November not shown because of limited sample size). However, the majority of historical year-to-year monthly comparisons showed relatively low *κ* values, indicating high variability (Fig. 4a–d). Therefore, contrary to our expectations (Prediction 5a), we found significantly lower year-to-year variability in the contemporary period than in the historical one during the months of December, January, and April (both *p*_*Dec*_ and *p*_*Jan*_ < 0.001, *p*_*Apr*_ = 0.036, Fig. 4a,b,e), while the year-to-year variability in February and March was not significantly different between the two periods (Fig. 4c,d). Similarly, we found this reduction in variability continued in the forecasted warming scenarios (counter to Prediction 5b), with the inter-annual variability within forecasted scenarios being significantly lower than in the historical period (Fig. 4).

**Figure 5:**
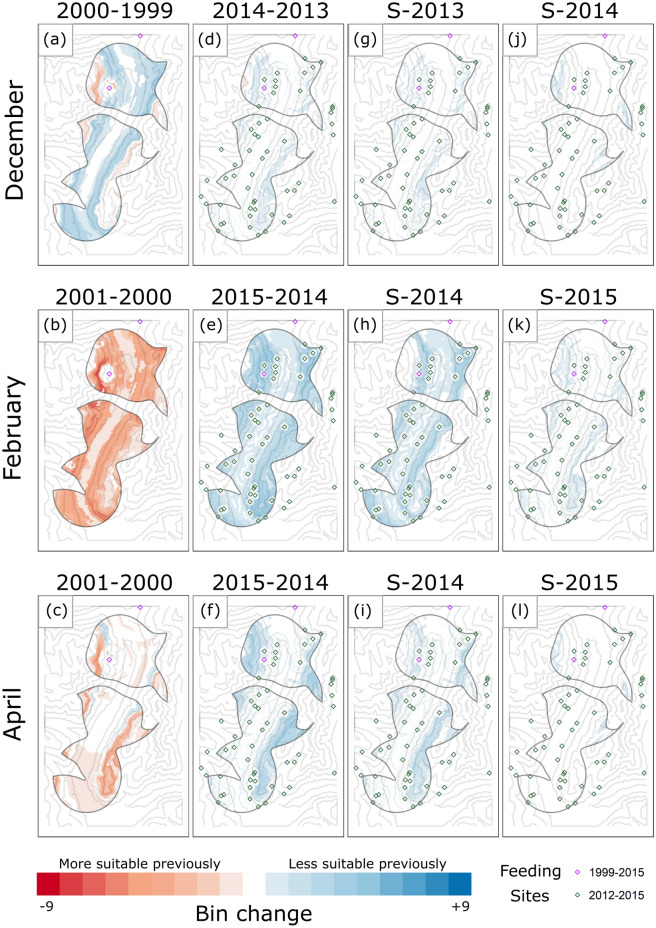
Maps of change in relative probability-of-use (*Rp*). Each map represents the subtraction of one binned *Rp* map from another (later map–earlier map). Pixel colors range from − 9, indicating extreme preference for the pixel in the earlier year, to + 9, extreme preference in a later year. The spatial resolution is 10 m, and the contour lines indicate 250 m changes in elevation, with lowest values in the central valley (see also Fig. 1). Panels (**a**–**f**) show subtractions within the historical period (**a**–**c**, low-snow year subtracted from high-snow year) and within the contemporary period (**d**–**f**, high-snow year subtracted from low-snow year); panels (**g**–**l**) show subtractions of contemporary years (high-snow: 2013/2014 winter; low-snow: 2014/2015 winter) from the same year predicted using the severe forecast scenario.

We have included subtraction maps comparing year-to-year relative *Rp*, in order to visualize the sole effect of snow variation on roe deer winter distribution under constant supplemental feeding intensity (Fig. 5). For both the historical and contemporary periods, when comparing a low-snow and high-snow winter (1999/2000 and 2000/2001, respectively, for the historical period; 2014/2015 and 2013/2014 for the contemporary period; see also Fig. S2.2), low-snow years exhibited an upslope shift of areas of high *Rp* out of the central valley. This was particularly true in the snowier months of mid-winter (February in Fig. 5, panels b and e respectively for the historical and contemporary periods). The comparison between the same two contemporary winters and the severe warming forecasted scenario confirmed this pattern is likely to continue in the future (Fig. 5g–l). Roe deer winter habitat suitability increased under predicted warming conditions, especially when compared to the particularly snowy winter of 2013/2014 (Fig. 5g–i), while smaller shifts in high-*Rp* areas were seen when comparing the forecast to the low-snow winter of 2014/2015 (Fig. 5j–l).

## Discussion

By making use of two tracking datasets separated by a decade and a half of anthropogenic change, we were able to empirically link an ungulate’s changing winter distribution to long-term ongoing and forecasted changes in abiotic constraints and anthropogenic resource availability. Specifically, our findings forecast a distributional upslope shift in relative probability of space use for an opportunistic, snow-constrained ungulate under current climate change, as a consequence of snow depth and snowpack changes. In our empirical setting, this response was made more complex by the attraction of animals to supplemental feeding sites, evidencing how snow depth and supplemental feeding can combine to modify the seasonal spatial distribution of ungulates and other mammals.

Resource selection models confirmed Predictions 1 and 2 in both study periods, showing a steep drop-off in relative probability-of-use at snow depths exceeding ~ 50 cm and at increasing distances from supplemental feeding sites. However, in the historical period, the attraction to supplemental feeding sites increased with snow depth (i.e., the relative probability-of-use was higher close to feeding sites in the case of deep snow), suggesting a mitigating behavioral response to the constraints associated with widespread heavy snowfall^3,6^. Conversely, in the contemporary period, these sites now attract roe deer almost irrespective of snow depth (i.e., the relative probability-of-use remained high close to feeding sites under almost any snow condition) (Prediction 3). As expected, we observed a substantial shift in roe deer’s relative probability of space use (i.e. distribution) from one decade to the next in response to an increased number and spread of supplemental feeding sites, together with the upwards shift of the snowline (Prediction 4). However, increased snow variability in contemporary years (given that the distribution of feeding sites is relatively static within this period) did not produce the expected increase in inter-annual *Rp* variability. Conversely, we found a decrease in inter-annual *Rp* variability in the early and late winter months (December, January, and April), and projections that this variability will continue decreasing in the forecasted scenarios (Prediction 5).

In the historical period, supplemental feeding sites mitigated the negative effects of deep snow, with higher *Rp* values extending in a radius of up to ~ 600–700 m from supplemental feeding sites (Fig. 3a). In the contemporary period, instead, higher *Rp* values extend only up to 200–300 m from feeding sites under the same snow conditions (Fig. 3b). Notably, *Rp* in close proximity to feeding sites remains high in the contemporary period under almost any snow conditions, showing a decreased mitigation effect, but stronger dependence on these artificial sources of food. These findings raise the question of the extent to which anthropogenic modifications of resource availability affect ungulate populations—particularly those facing particularly limiting winter conditions^27^. Potential drawbacks of supplementing feeding include, but are not limited to, transforming feeding sites into “ecological traps”^28^, where disease transmission^29,30^ or competition-induced stress increases^15,31^ can severely affect population dynamics. Behavioral responses may enable animals to adapt to anthropogenic changes^32–34^, but in many cases, these responses may be insufficient and even maladaptive^28,35^. For instance, supplemental feeding sites may make animals more susceptible to harsh snowfall in the studied population, as they congregate in areas they would normally avoid in the winter and lose the capacity to rely on natural food resources^15,36^, potentially with negative effects on their health^37,38^. Anecdotally, five of our collared roe deer died in one week in March 2014 following an intense snowfall, despite sustained use of feeding sites. Increased predation risk due to artificially elevated local density of roe deer around feeding sites may prove another unanticipated drawback of the practice^39^. While this effect is currently not a major issue in our study system (only brown bears, *Ursus arctos*, may predate on adult roe deer, but they hibernate in winter), it could be increasingly be the case as large carnivores expand through Europe^40,41^. Thus, even as the positive effects of supplemental feeding practice remain controversial^42^, there is rising concern around the pitfalls of the practice and its far-reaching effects on behavior^43–45^.

While the increase in supplemental feeding sites played the largest role in driving inter-decadal shifts in space use, snow variability exerted a fundamental role in shaping year-to-year variation in relative probability of space use. Remarkably, we observed the high variability expected in the contemporary period (Prediction 5a) only during the central months of the winter (February–March), while in the historical period deer exhibited relatively high space use variability all winter long. The low variability in contemporary space use patterns detected for the months of December, January, and April (Fig. 4) was likely due to scarce-to-absent snowfall in these months in contemporary years. Therefore, milder conditions at the beginning and end of winter reduced the constraint of snow cover on roe deer winter distribution and allowed relative probability-of-use to remain highly consistent from year to year during these months. Conversely, the unexpected variability in historical space use is arguably the standard variability to be expected from year-to-year changes in snow patterns, and should be contrasted with the climate change-driven lowered year-to-year variability of the contemporary period and forecasted scenarios. These patterns presage the speed with which climate-driven changes in snow cover and related animal behavioral responses are happening. The continued upward shift of the snowline along the elevation gradient in our climate change forecasts (Prediction 5b) confirms this trend and in turn forecasts substantial upslope expansion of suitable winter habitat for roe deer in the Alps.

Our work empirically supports the theoretically predicted effects of a decrease in snow coverage in the Northern Hemisphere^46^ on distribution range shifts and movement patterns of varied mammal species^47^. Such changes have been found to affect the probability of seasonal migration from summer to winter ranges in several ungulate species^8,48,49^, with deep snowpacks driving moose (*Alces alces*) to modify their fine-scale movements to search for conifer patches within mixed stands^50^, and Sitka deer (*Odocoileus hemionus sitkensis*) selecting for different successional stages of forest within their winter range according to snow depth variability^51^.

This study expands these findings by providing rare empirical evidence of the interplay between anthropogenic changes in climate and food supply^32^ in driving behavioral responses in a large herbivore at the altitudinal limit of its distribution. Moreover, it examines behavioral responses at the inter-decadal scale at which changes in the snowpack become measurable and sustained. Although our analysis is confined to the alpine watershed in which our study was executed, it is applicable to a broad guild of temperate and arctic ungulates. More generally, it provides an example of the general effects of interacting pressures on space use patterns at the range limits of all species^11,27^. Under predicted changes, species may change their distribution^47^, undergo local extirpations^52^, or adapt within their current range, as observed in this study.

Therefore, the topic is of great relevance—particularly to ungulates, as their proximate behavioral responses will likely have cascading effects on local ecosystems^27^, e.g. through changing browsing pressure, nitrogen cycling, and seed dispersal^53^, or through inter-specific competition^54^. We therefore encourage other animal ecologists to make use of localized climatological and movement data to shed light on these and other important questions linking environmental change with space use trends. Further, future research should consider the demographic consequences of these ongoing changes, particularly by linking expanding habitat suitability to population dynamics at the range limits of snow-constrained species under climate change.

## Methods

### Study area

All research was carried out in a 400 km^2^ mountainous area ranging in altitude from 400 to 3500 m in the north-eastern Italian Alps (Val Rendena and Valli Giudicarie, Autonomous Province of Trento, Italy; see Supplement S1 for details on climate, flora, and fauna). Over the study period, the area saw an increase in supplemental feeding for roe deer, from 20 to 95 actively managed sites (Fig. 1), due to modifications in hunting management regulations. The overall population density of roe deer in the area has remained relatively low and constant throughout the last two decades (i.e., between three and five deer/100 ha)^55,56^.

### Movement data

The dataset of contemporary roe deer relocations was collected from December 18th 2012 to April 20th 2015, for a total of 33,399 winter GPS relocations (1 Nov–30 Apr; one fix every 3 h) from 23 roe deer (9 males, 14 females). Animals were captured using box traps and drop nets during two field campaigns in the winters of 2012–2013 and 2013–2014 and outfitted with GPS-GSM radio-collars (VECTRONIC Aerospace GmbH).

The dataset of historical roe deer relocations was collected from March 7th 1999 to April 30th 2002, for a total of 4529 winter VHF radio-collar relocations (one fix per animal per day^22^) from 32 roe deer (10 males, 22 females), captured using box traps and drop nets, then outfitted with VHF transmitters (Televilt International AB).

All animal handling practices for capturing and marking roe deer complied with Italian laws on animal welfare and were approved by the Wildlife Committee of the Autonomous Province of Trento.

### Environmental data

We used the GEOtop 2.0 Hydrological Model^23^ (hereafter “GEOtop”) to generate a 25-year long time series (1989–2015) of daily variation in snow depth at a spatial resolution of 100 m. GEOtop is a water- and energy-balance model that produces snow depth area maps from meteorological data, taking into account snow melting processes across the landscape (see Supplement S2 for details). We also used GEOtop in conjunction with a regional climate model (COSMO-CLM)^57^ to generate two further 25-year long time series (hereafter “intermediate” and “severe” scenarios) using forecasts of radiative forcing from greenhouse gas emission scenarios of differing severity (IPCC RCP4.5^25^ and RCP8.5^26^). We assessed the accuracy of GEOtop output for our study area by comparing predicted snow depth data to empirically measured snow depth measurements collected from 2012 to 2015^20^, and we applied a correction coefficient to remedy slight overprediction of GEOtop in forested areas (see Supplement S2). For each generated timeseries separately, we obtained monthly snow depth maps for the entire study area by averaging the daily GEOtop snow depth values over each winter month (November–April).

We derived canopy cover maps for the historical and contemporary periods, respectively, from CORINE 2000 and 2012 land cover maps (European Environment Agency 2000 and 2012, available at https://land.copernicus.eu/pan-european/corine-land-cover; hereafter "CLC 2000" and "CLC 2012"). For both maps, we ranked broad-leaf forest, coniferous forest, and mixed forest land classes as “closed” canopy, and all other classes as “open”.

Finally, we gathered supplemental feeding site locations from local hunters and wildlife managers. We used ArcGIS software (ESRI, Redlands, CA, USA) to compute two maps (one for each study period) of 3D path-distance to the nearest supplemental feeding site.

### Predicting habitat selection

We estimated two resource selection functions (RSFs) to predict roe deer space use patterns across the study area in the historical and in the contemporary periods. RSFs are functions proportional to the probability a given animal will select a resource unit. They are commonly used to understand species’ distributions and space use by characterizing the relative probability-of-use of a given site as a log-linear product of its characteristics and appropriate selection or avoidance coefficients (see Eq. 1 below)^58^. We calculated these RSF coefficients by fitting a logistic regression to telemetry-derived locations *used* by roe deer (case = 1) between 1999 and 2002 (historical RSF, “RSF_h_”) or 2012–2015 (contemporary RSF, “RSF_c_”) and to *available* locations (case = 0)^59^. After confirming the consistency between daily and nocturnal versions of the GPS-based RSF_c_ (see Supplement S4), we retained only GPS relocations between sunrise and sunset for the RSF_c_ used in the analysis (daylight deduced using the *suncalc* R package^60^). Limitation to daylight hours was done to permit strict comparison of the VHF- (historical) and GPS-based (contemporary) models, since VHF data had been only gathered during daylight hours. For both RSFs, we randomly selected one used point per animal per day (final used sample: GPS 4625 points, VHF 4013 points) in order to reduce autocorrelation between points and to further strengthen the comparison between VHF- and GPS-based datasets, given their different temporal resolutions. For both RSFs, available points were randomly sampled at a 1:1 ratio to used points^61^ within 99% kernel density polygons (bivariate normal with reference bandwidth) of all VHF and GPS roe deer locations, respectively.

We estimated the historical (RSF_h_) and contemporary (RSF_c_) relative probability of space use (used/available) at a monthly scale, as a function of (i) monthly averages of GEOtop-derived snow depth (*SD*) (Prediction 1), (ii) the reciprocal of the distance between the location and the nearest supplemental feeding site (*1/Dist*_*FS*_, “proximity”) (Prediction 2), and (iii) the interaction between these two covariates (*SD/Dist*_*FS*_) (Prediction 3), while controlling for canopy cover as a fixed effect (*CC*). We chose *1/Dist*_*FS*_ rather than the distance to supplemental feeding sites because, when predicting over large areas, an ever-decreasing linear effect of supplemental feeding sites on habitat selection becomes biologically unrealistic beyond perception limits. All predictions of relative probability-of-use (*Rp*) were based on a linear scaling (from 0 to 1, Eq. 2) of the log-linear form in Eq. 1 (typical for RSFs^59^):

1 $$Rp= LinScale\left(\mathrm{exp}\left[{\widehat{\beta }}_{1}\left(CC\right)+ {\widehat{\beta }}_{2}\left(SD\right)+ {\widehat{\beta }}_{3}\left(\frac{1}{Dis{t}_{FS}}\right)+ {\widehat{\beta }}_{4}\left(\frac{SD}{Dis{t}_{FS}}\right)\right]\right)$$

where

2 $$LinScale\left({x}_{i}\right)=\frac{ {x}_{i}-\mathrm{min}\left(x\right)}{\mathrm{max}\left(x\right)-\mathrm{min}\left(x\right)}$$

We evaluated the reliability of both RSF_h_ and RSF_c_ using Receiver Operating Characteristics (ROC) curves and k-fold cross-validation^59^ with Kendall’s tau (*τ*) coefficients^62^ (which are typically more robust than Spearman’s rho). In k-fold cross-validation, data are partitioned into k equal subsets (5 in this study), and each subset is used to define a model. *τ* coefficients measure the correlation between rankings of model predictions in the remaining data and area-adjusted frequencies of observed habitat use^59^. We also used RSF_h_ to predict *Rp* for 1999–2002 and RSF_c_ to predict *Rp* for 1999–2002 and 2012–2015, further using *τ* coefficients to spatially and temporally validate the models’ ability to predict actual space use (Supplement S5). We thus considered significant τ coefficients produced within a model’s respective study period to be a spatial validation of that model (spatial RSF_h_ *τ* in 1999–2002, spatial RSF_c_ *τ* in 2012–2015) and significant *τ* coefficients produced in 1999–2002 for RSFc to be a temporal validation of the contemporary model’s predictive ability (temporal RSF_c_).

Finally, we also forecasted *Rp* using RSF_c_ and snow depths estimates from both forecast scenarios of climate change (*intermediate scenario* = GEOtop monthly average outputs 2012–2015 with RCP4.5 correction + CLC 2012 + distribution of supplemental feeding sites in 2012–2015; *severe scenario* = GEOtop monthly average outputs 2012–2015 with RCP8.5 correction + CLC 2012 + distribution of supplemental feeding sites in 2012–2015).

### Evaluating temporal changes in patterns of space use

We used weighted Kappa coefficients^24^ to examine how changes in snow patterns and supplemental feeding site distribution have affected variability in spatial patterns of *Rp* of identical months between periods (“inter-decadal”, Prediction 4) and within a study period (“year-to-year”, Prediction 5). Kappa coefficients (*κ*) provide a proportion of agreement (“similarity”) between two maps’ binned *Rp* values (pixel-by-pixel), accounting for the probability of overlap between randomly-distributed *Rp* values (e.g., 0.1 * 0.1 = 1% of the lowest 10% of *Rp* values should overlap by chance in any two maps). *κ* coefficients take on a value of 1 for perfect agreement and values near 0 for agreement no larger than expected by chance^24^, thus providing a robust means of comparison between entire spatial surfaces.

For each winter month (Nov–Apr) we extracted monthly *Rp* values (generated with the corresponding period’s RSF) for 1000 random locations within the intersection of both study periods’ 99% kernel polygons and binned these into 10 quantiles established from the pooled *Rp* values generated from RSF_c_ across the entire study. We then used these binned values to compute a *κ* coefficient, weighted by the degree of rank disagreement^63^. For a given winter month, we thus calculated a *κ* coefficient for each possible annual pairwise combination (a) within the historical study period (“intra_h_”), (b) within the contemporary study period (“intra_c_”), (c) between the two study periods (“inter-decadal”), (d) within the intermediate scenario (“intra_i_”), and (e) within the severe scenario (“intra_s_”). For each month, we used a one-way ANOVA and post-hoc Tukey tests to evaluate the significance of differences between the *κ* coefficients of these five categories.

Finally, we created subtraction maps to visually represent the comparisons captured by the *κ* coefficients within each period (i.e. year-to-year), thus keeping fixed the availability of supplemental feeding sites. We chose three months to represent different parts of the winter: December (early winter), February (mid-winter), and April (late winter). For each month, we firstly created maps of *Rp*, binned into the same 10 quantiles developed for calculating the *κ* coefficients. Then, we computed four subtraction maps in order to visually illustrate the areas of highest difference in relative probability-of-use. Specifically, both for the historical study period (intra_h_) and for the contemporary one (intra_c_), we compared a dry and a snowy winter (1999/2000 and 2000/2001, respectively, for the historical period; 2014/2015 and 2013/2014 for the contemporary period; information on snow characteristics in 2014 and 2015 were extracted from GEOtop layer, see also Fig. S2.2). We also created subtraction maps to compare both contemporary winters with the maps predicted under the most severe warming scenario. In all cases, the earlier map was subtracted from the later map.

Kernel polygons and path distances between pixels were generated in ArcGIS spatial software (ESRI, Redlands, CA, USA). All other statistical analyses and spatial analyses were conducted using R 3.4.3 (key packages used: *irr*^64^, *ResourceSelection*^65^, *rgdal*^66^, *Kendall*^67^).

## Supplementary Information

Supplementary Informations.

## Data Availability

The data are available in the Eurodeer repository (http://www.eurodeer.org), accessible following user registration. The datasets are also available in Zenodo at10.5281/zenodo.4637674. (Bright Ross et al^68^, 10.5281/zenodo.4637674).
