# Supplementary material for: Climate change and anthropogenic food manipulation interact in shifting the distribution of a large herbivore at its altitudinal range limit
Source: Sci Rep. 2021 Apr 7;11:7600. doi: 10.1038/s41598-021-86720-2 (PMC8027592; doi:10.1038/s41598-021-86720-2)
Supplement: Supplementary file 1 — Supplementary Informations. [file 41598_2021_86720_MOESM1_ESM.docx]

**Supplementary material for “Climate change and anthropogenic food manipulation interact in shifting the distribution of a large herbivore at its altitudinal range limit”**

**List of Authors:** Julius G. Bright Ross, Wibke Peters, Federico Ossi, Paul Moorcroft, Emanuele Cordano, Emanuele Eccel, Filippo Bianchini, Maurizio Ramanzin, and Francesca Cagnacci

**Supplement S1: Characterization of study area and climatic trends in Trentino**

The study area comprises rugged mountainous terrain ranging from 400 m to 3500 m in elevation above sea level, largely covered by a mix of coniferous and broadleaf forest comprised of pine (*Pinus* spp.), spruce (*Picea* spp.), larch (*Larix decidua*), common beech (*Fagus sylvatica*) and European ash (*Fraxinus excelsior*). The elevation of the tree-line depends on localized topography, but lies at a mean elevation of 1800 m, above which shrubs such as mountain ash, rhododendron and *Vaccinium* spp. prevail. Alpine meadows are dominant between 2000-2400 m, representing the elevational limit of the vegetation line. Roe deer are present at a density of 2-5 individuals/km^2^, a number that has not changed over the course of the study^1,2^. Chamois (*Rupricapra rupricapra*), red deer (*Cervus elaphus*), mouflon (*Ovis musimon*), and ibex (*Capra ibex*) are also present, while red foxes (*Vulpes vulpes*) and a reintroduced population of brown bears (*Ursus arctos*) were the area’s only non-human large predators at the time of the study. Hunting occurs throughout the area from September through December with selective quotas.

The study area is characterized by a typical alpine-continental climate, due to its rugged topography and elevation. Based on data from 1990 to 2013, average yearly rainfall in the area is around 1100 mm (winter: 198 ± 104 mm), while average monthly temperature ranges from -1 °C in December to 18 °C in July (data source: www.meteotrentino.it).

A long-term analysis of seasonal precipitation patterns in Trentino^3^ showed that, over the past 90 years, winter precipitation patterns in the region have been moderately variable, with frequent and repeated periods of slightly positive or slightly negative (mostly non-significant) patterns (Fig. S1.1b). The same study, however, found that regional temperatures have risen steadily since the middle of the past century (Fig. S1.1a). Accordingly, an analysis of winter temperature anomalies at a meteorological station located within the study area (Tione di Trento) showed an increase of 1.5 °C in winter (Dec-Feb) average temperature over the past three decades (Fig. S1.2a^4^). These changes in temperature and precipitation drove a marked decrease in snow cover duration over the decades preceding the beginning of our study (Fig. S1.2b), especially at elevations below 1500 m^5^.


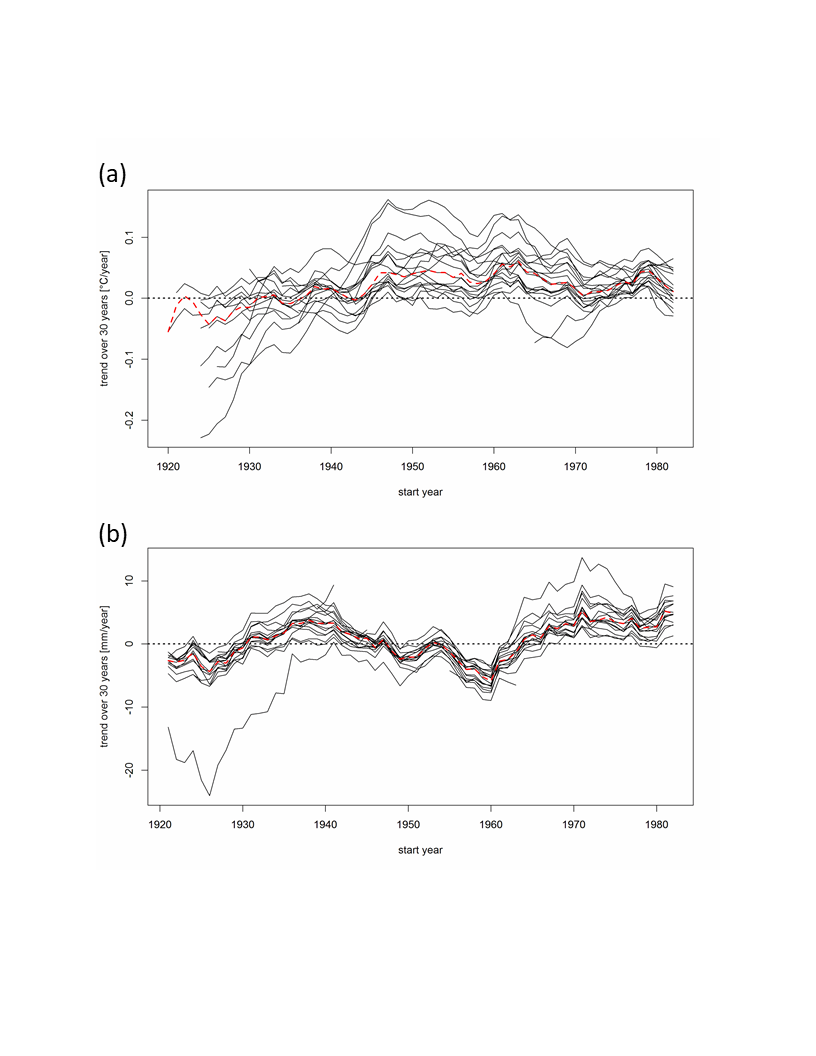


Figure S1.1: 30-year moving trends for winter (Dec-Feb) (a) temperature, and (b) precipitation. Each black continuous line represents a weather station (n = 13), while the red dotted line denotes the year-by-year median of these; for any year on the x-axis, the corresponding value on a line represents the 30-year moving trend of the following 30 years for that station, meaning the x-axis captures information up to 2012. Reproduced with permission from Eccel et al. 2013^3^.


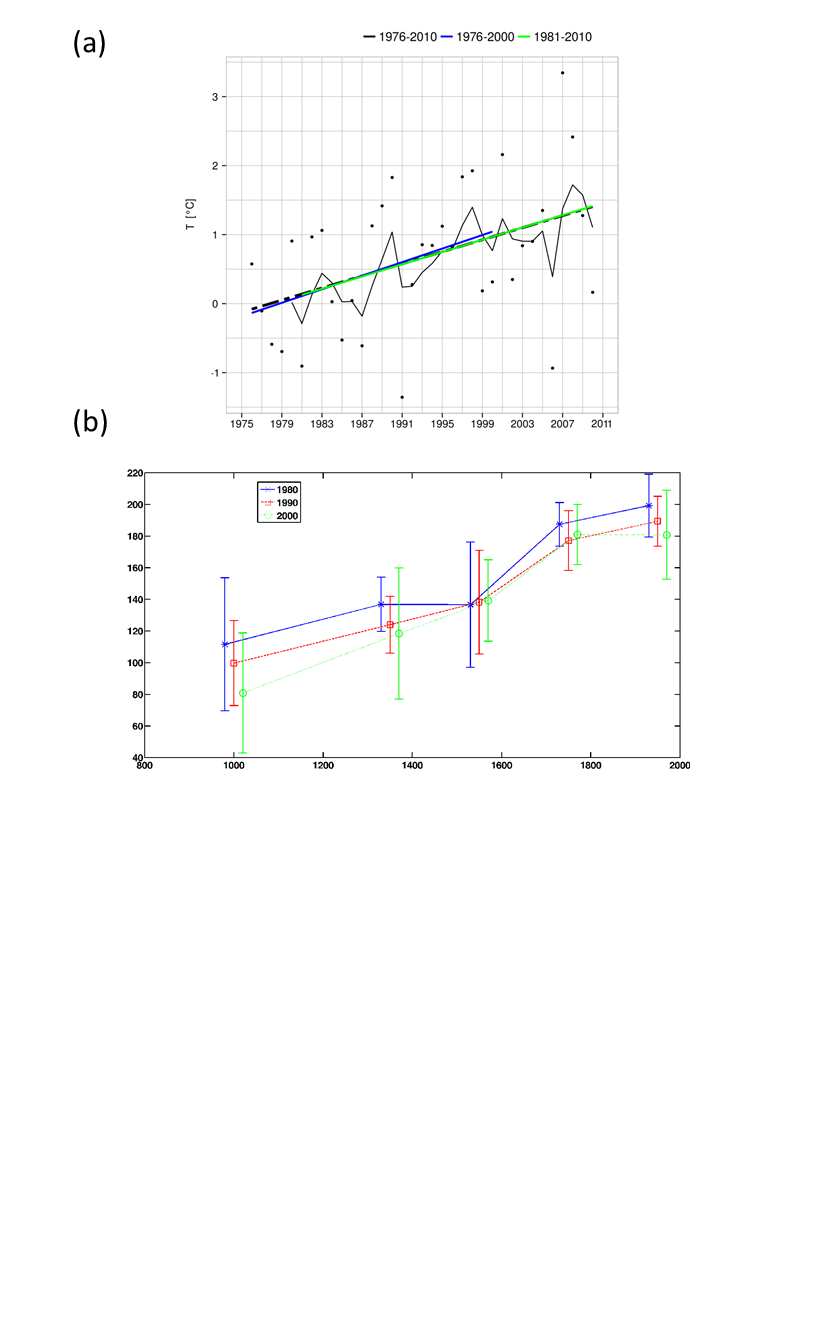


Figure S1.2: Winter conditions over the past three decades in our study area, showing: (a) average winter temperature (Dec-Feb) of Tione weather station from 1975 to 2010 (data used from Di Piazza et al. 2012^4^ with permission to create new figure) and (b) snow cover duration (days) as a function of elevation for each of the three past decades (reproduced with permission from Bellin et al. 2015^5^). Lines represent trend for a corresponding period: in a), black, dashed: whole period; blue: 1975-2000; green: 1981-2010; in b), blue: 1981-1990; red: 1991-2000; green: 2001-2010. Error bars in b) denote variation within the 14 snow fields used to create the plot.

**Supplement S2: GEOtop 2.0 Hydrological Model to predict snow depth: prediction maps, empirical validation, and snow depth mapping across the study area**

*GEOtop Model*

The GEOtop 2.0 Hydrological Model (hereafter “GEOtop”) is a distributed grid-based simulator that represents the heat and water budgets at and below the soil surface and in the low atmosphere (Bertoldi et al., 2014; Endrizzi et al., 2014). GEOtop takes into account the effects of topography on the interactions between energy balance (radiation and turbulent thermal fluxes) and the water cycle (precipitation, snow melting, etc.). By doing so, it can estimate the geospatial distribution of soil moisture and snow depth used in this study, given observed weather station data (precipitation, air temperature, solar short-wave global radiation, wind velocity, and air pressure), topographical data (a Digital Terrain Model—DTM), and land-use maps. In particular, GEOtop incorporates a robust physically-based module for snow melting processes (Endrizzi and Marsh, 2010), making it an ideal resource for this study. Further details of the GEOtop Hydrological Model and on its development are available at www.geotop.org.

*Time series generation*

This study built on daily average snow depth maps at a 100 m spatial resolution. These outputs were generated using meteorological data from June 1^st^, 1989 to June 30^th^, 2015 (precipitation, air temperature, solar short-wave global radiation, wind velocity, and air pressure) from 23 stations in and around Val Rendena and Valli Giudicarie, operated by Meteotrentino (www.meteotrentino.it) and Fondazione Edmund Mach (www.fmach.it). Using these data, GEOtop was used to generate snow depth outputs for every day in every winter month (November – April) over the entire Val Rendena/Valli Giudicarie watershed.

GEOtop was also used in conjunction with outputs from the COSMO-CLM (Rockel et al., 2008) regional climate model (RCM) to generate future scenarios of snow depth in our study area. The RCM was applied over Italy, employing a spatial resolution of 0.0715° (approx. 8 km). Two separate simulations were performed, employing the IPCC’s “RCP4.5” and “RCP8.5” representative concentration pathway (RCP) greenhouse gas (GHG) concentrations. RCP4.5 represents stabilization in global GHG emissions after 2070, with a global radiative balance 4.5 W/m^2^ higher than pre-industrial values. RCP8.5 is characterized by a rapidly increasing GHG concentration, increasing radiative balance by 8.5 W/m^2^. Details on the simulation’s performance can be found in Bucchignani et al. (2016).

RCM outputs were downscaled to our study area at weather station level, using Tione as the reference for the area. Downscaling was achieved through a bias correction by statistical downscaling, based on a quantile mapping technique (Zollo et al., 2015). Due to the lack of projections of meteorological quantities other than temperature and precipitation, the hourly datasets used in the GEOtop snow depth simulations were created by applying monthly correction coefficients to the existing instrumental series calculated from these downscaled projections. The periods used to compare present to future scenarios were 1996-2015 and 1946-2065, respectively. The temperature bias of the two projection scenarios was applied additively while precipitation bias was applied as a multiplicative correction.

*Empirical validation of GEOtop timeseries and correction factor design*

We evaluated the GEOtop output data using snow depth data empirically collected during the field campaigns of 2012-2013 and 2013-2014 (2012-2013: Ossi et al., 2015; 2013-2014: Bianchini and Ossi, unpublished work). Specifically, for each snow sampling site (n = 622), we computed a new snow parameter denoted “adjusted snow depth” (ASD; ASD = average + standard deviation of the sixteen snow depth measurements taken at that plot, to capture the heterogeneity of measurements in sampling plots within the forest). We compared the ASD of each plot to GEOtop output. To do so, we subtracted ASD at each sampling plot from the corresponding GEOtop output value on that day (GEOtop Snow Depth, GSD) to produce a new metric, termed “Δsnow” (Δsnow = GSD – ASD). We then binned all Δsnow values into 10% quantiles (Tab. S2.1).

We observed that Δsnow differed substantially between forested plots and those falling in open areas (Fig. S2.1). We argue this was most likely due to the coarse spatial resolution used for GEOtop simulations, which did not account for a “snow filtering” effect arising from canopy cover. We therefore derived a correction factor for GEOtop values falling in wooded areas. To do so, we used a linear model to contrast the measured ASD of open and forested sampling sites, keeping other environmental characteristics (elevation, slope, and aspect) fixed. We obtained a coefficient of 0.46 (p < 1.19e-13), denoting the modelled difference in empirical snow depth between forested and open sites. We applied this coefficient to those GEOtop values falling in woody areas, and re-computed the Δsnow value for each plot. After binning Δsnow into 10% quantiles, we found that the fit between GEOtop output and empirical data improved, with 80% of Δsnow values ranging between -238 and 298 mm (Tab. S2.1). Therefore, we applied this correction coefficient to the monthly GEOtop snow depth maps used to calculate our predictive maps of roe deer habitat selection.


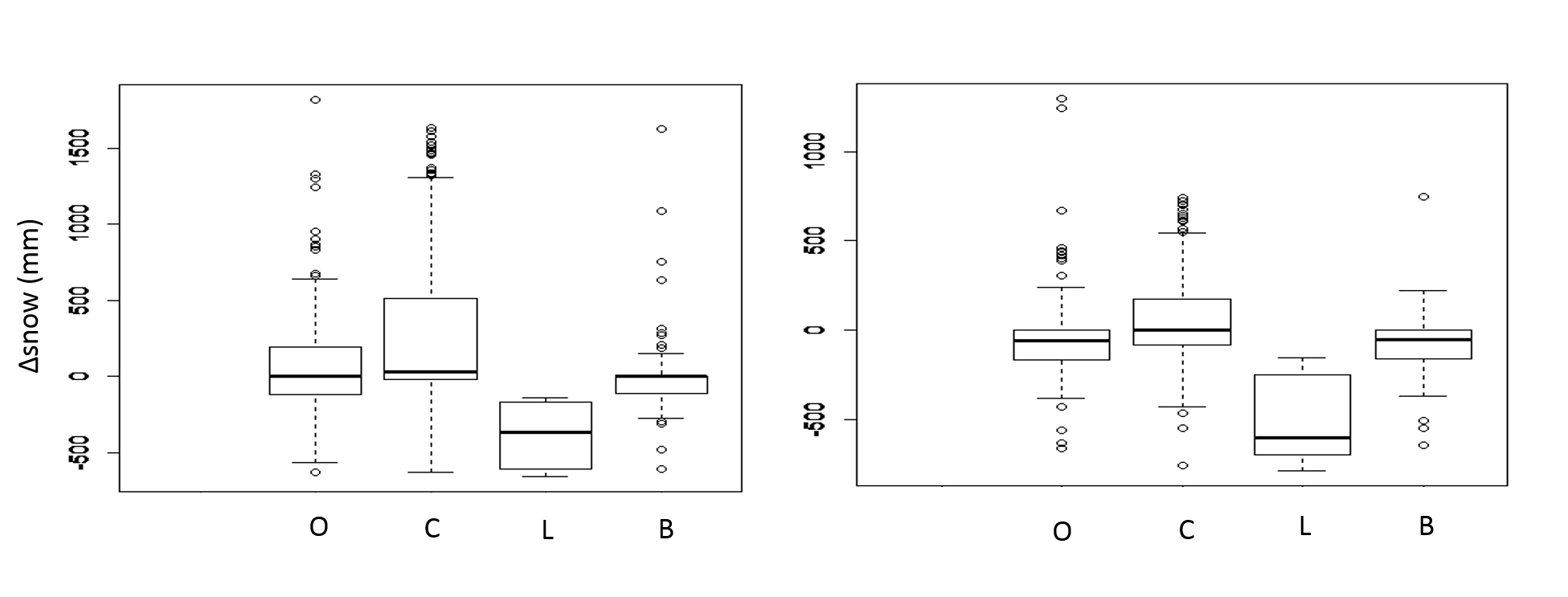


Figure S2.1 - Comparison of Δsnow values (in millimeters) before (left) and after (right) the correction coefficient application for forested sampling points, for different environmental categories (O = open; C = conifer; L = larch wood; B = broad-leaved forest)

| **Δsnow \ quantile** | 0 | 10 | 20 | 30 | 40 | 50 | 60 | 70 | 80 | 90 | 100 |
| --- | --- | --- | --- | --- | --- | --- | --- | --- | --- | --- | --- |
| Pre-correction | -655 | -184 | -113 | -49 | 0 | 0 | 10 | 145 | 357 | 748 | 1817 |
| Post-correction | -788 | -238 | -155 | -104 | -59 | 0 | 0 | 4 | 118 | 298 | 1298 |

Table S2.1 - Δsnow distribution across the 622 sampled sites, before (first raw) and after (second raw) applying correction coefficient to GEOtop values. Values are provided in millimeters.

*Snow depth mapping across decades and throughout roe deer study area*

We averaged GEOtop daily values into monthly maps of snow depth. We then binned pixels within the union of 99% kernel polygons constructed for each period’s animal relocations (see main text) on these maps into elevational bands for each month covered by the study, to examine ongoing changes in patterns of snow depth (Fig. S2.2). We then applied the correction coefficient to the monthly average snow depth maps to be used with RSFs to predict relative probability of use (see main text).


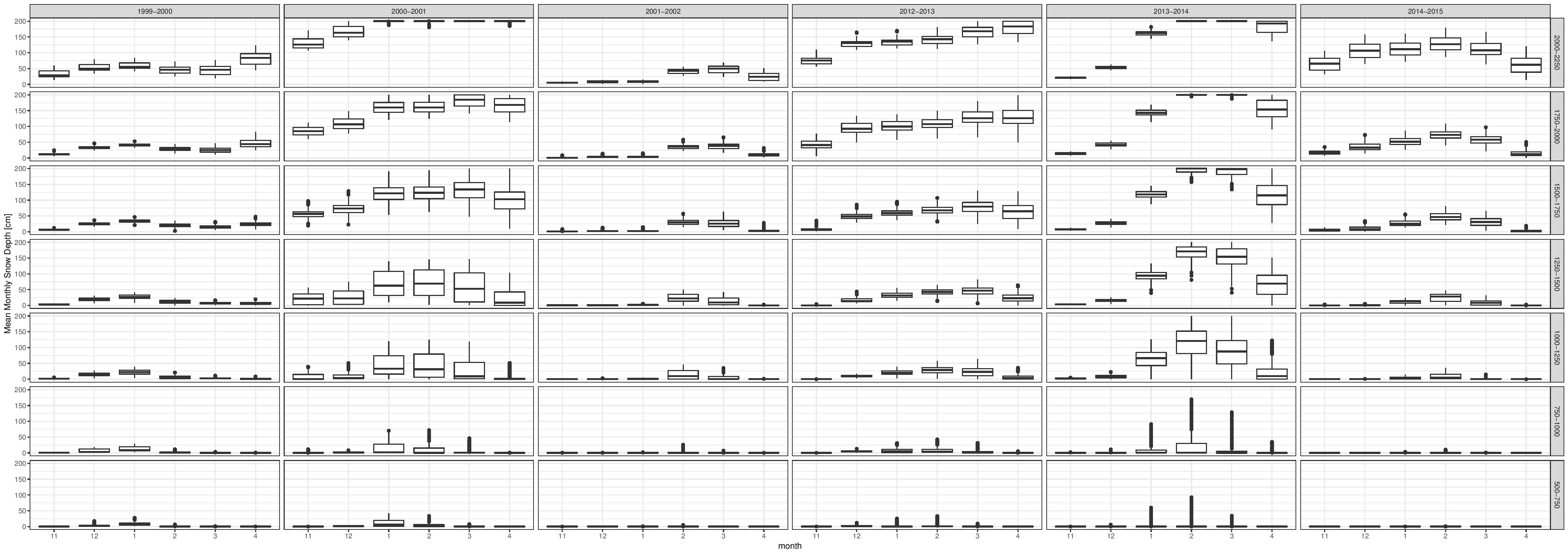


Figure S2.2 – Snow depth classified by month and elevation belts in the area covered by the roe deer movement studies across the historical (1999-2002) and contemporary (2012-2015) periods, as derived by the GEOtop model.

**Supplement S3: Additional figures**


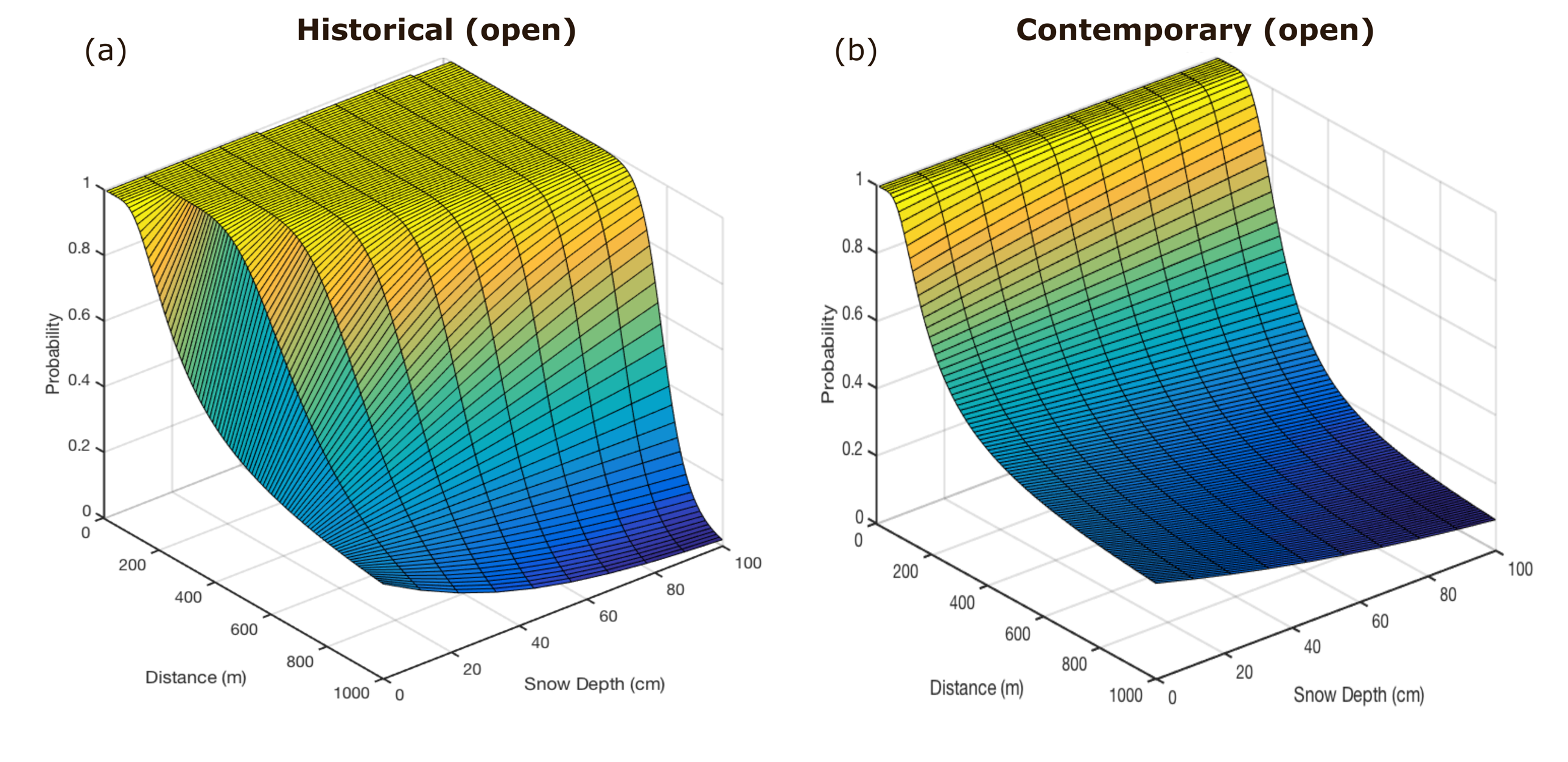


Figure S3.1: 3D prediction plots of relative probability of use of non-forested habitats in the two periods (1999-2002: (a); 2012-2015: (b)) covered by the analysis.


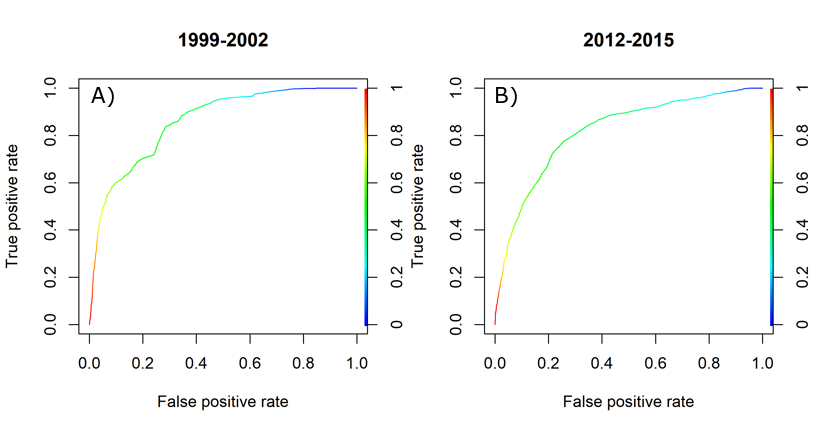


Figure S3.2: Receiver operating characteristics (ROC) curves for a) historical and b) contemporary RSF models.

**Supplement S4: Comparison between nocturnal and diurnal GPS-based resource selection functions (RSF_c_)**

Roe deer commonly exhibit a circadian alternation of behavioral modules, with peaks of activity concentrated in crepuscular and night hours^13^, although in wintertime it is not uncommon to observe an increase in activity during the day^14^. Since we built the RSF_c_ model based solely on daily GPS locations to be consistent with RSF_h_ (for which nocturnal VHF locations were not available), we investigated whether this selection biased the coefficients of RSF_c_. To do so, we constructed an analogous RSF_c_ based on nighttime GPS locations, and we compared the output of the daytime and nighttime RSF_c_ models. We found a high consistency between the two RSFs (Tab. S4.1 and Fig. S4.1), excepting only selection for canopy cover. During nighttime, roe deer’s selection for canopy cover was still positive, but not significant, probably because roe deer tend to use open areas more during crepuscular and night hours, as predation at these times is less likely^15^. Since the main target of this analysis was to explore the variation of roe deer spatial behavior in light of changing snow cover and supplemental feeding, we believe that the different magnitudes of the effect of canopy cover during the day and at night (implemented in any case as a control factor) should not bias the analyses. We therefore concluded that constructing our RSF_c_ model using daytime GPS relocations does not bias the output of the analyses and the consistency of the pattern described.

| **Covariate** | **Daily** | **Nocturnal** |
| --- | --- | --- |
| Canopy cover (1 = forested) | 8.033e-1*** | 4 4.502e-02.502e- |
| Snow depth (cm) | -1.796e-3*** | -1.702e-3*** |
| 1/Distance to feeding site (1/m) | 3.508e+2*** | 3.602e+2*** |
| Snow depth/distance to feeding site | 4.282 e-1*** | 2.798 e-1*** |

Table S4.1 – Coefficients of models constructed using either daily or nocturnal GPS used points. Asterisks denote significance of term in model (*** = p < 0.001).

*
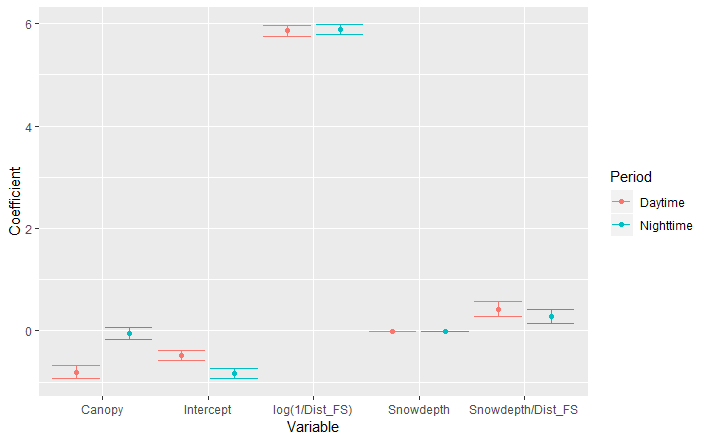
*

Figure S4.1 – Whiskers plot comparing the values of the coefficients of the daytime (red) and nighttime (blue) GPS-based RSFc. The bars denote 95% confidence intervals associated with each coefficient. Note that due to the different scale of the 1/Dist_FS_ covariate, we rescaled its corresponding β by taking its natural log.

Supplement S5: Spatial and temporal validation of the Historical and Contemporary Resource Selection Functions

For spatial validation of RSF_h_ and RSF_c_, we applied Eq. 1 (see main text, Methods section) across the entire study area for each winter month and rescaled predictions from 0 to 1 to produce maps of *Rp* (historical, predicted using RSF_h_ = GEOtop monthly average outputs 1999-2002 + CLC 2000 + distribution of supplemental feeding sites in 1999-2002; contemporary, predicted using RSF_c_ = GEOtop monthly average outputs 2012-2015 + CLC 2012 + distribution of supplemental feeding sites in 2012-2015). For temporal validation, we applied the same procedure, but historical maps of *Rp* were predicted using RSF_c_. For each predicted monthly map, we then extracted values of *Rp* underlying a) used points and b) 1,000 new available points that we randomly generated within the population home range polygon of each respective study period. We used the pooled used and available *Rp* values across the entire study to derive 10 quantiles. Within each month, we sorted used and available *Rp* values into these quantiles and we used Kendall’s tau rank correlation coefficients to examine whether the ratio of used-to-available points increased in higher quantiles.

**Supplementary References**

1. Carnevali, L., Pedrotti, L., Riga, F. & Toso, S. *Banca dati ungulati: Status, distribuzione, consistenza, gestione e prelievo venatorio delle popolazioni di ungulati in Italia. Rapporto 2001-2005*. *Biologia e Conservazione della Fauna* **117,** (2009).

2. Provincia Autonoma di Trento. *Analisi delle consistenze e dei prelievi di ungulati, tetraonidi e coturnice. Stagione Venatoria 2018.* (2018).

3. Eccel, E. *Progetto CLITRE.100 - elaborazione ed analisi delle serie secolari in Trentino, scenari di evoluzione climatica, progetto di Atlante Climatico Trentino - relazione conclusiva*. (2013).

4. Di Piazza, A. & Eccel, E. *Analisi di serie giornaliere di temperatura e precipitazione in Trentino nel periodo 1958-2010*. (2012).

5. Bellin, A., Marcolini, G. & Chiogna, G. *Omogeneizzazione ricostruzione delle serie storiche dei dati neve disponibili in Trentino*. (2015).

6. Bertoldi, G. *et al.* Process-based modeling of vegetation dynamics, snow, evapotranspiration and soil moisture patterns in an alpine catchment. *Geophys. Res. Abstr.* **16,** (2014).

7. Endrizzi, S., Gruber, S., Dall’Amico, M. & Rigon, R. GEOtop 2.0: simulating the combined energy and water balance at and below the land surface accounting for soil freezing, snow cover and terrain effects. *Geosci. Model Dev.* **7,** 2831–2857 (2014).

8. Endrizzi, S. & Marsh, P. Observations and modeling of turbulent fluxes during melt at the shrub-tundra transition zone 1: point scale variations. *Hydrol. Res.* **41,** 471–491 (2010).

9. Rockel, B., Will, A. & Hense, A. The regional climate model COSMO-CLM (CCLM). *Meteorol. Zeitschrift* **17,** 347–348 (2008).

10. Bucchignani, E., Montesarchio, M., Zollo, A. L. & Mercogliano, P. High-resolution climate simulations with COSMO-CLM over Italy: performance evaluation and climate projections for the 21st century. *Int. J. Climatol.* **36,** 735–756 (2016).

11. Zollo, A. L., Turco, M. & Mercogliano, P. Assessment of hybrid downscaling techniques for precipitation over the Po river basin. in *Engineering Geology for Society and Territory - Volume I* 193–197 (2015).

12. Ossi, F., Gaillard, J. M., Hebblewhite, M. & Cagnacci, F. Snow sinking depth and forest canopy drive winter resource selection more than supplemental feeding in an alpine population of roe deer. *Eur. J. Wildl. Res.* **61,** 111–124 (2015).

13. Wallach, A. D., Shanas, U. & Inbar, M. Feeding activity and dietary composition of roe deer at the southern edge of their range. *Eur. J. Wildl. Res.* **56,** 1–9 (2010).

14. Pagon, N. *et al.* Seasonal variation of activity patterns in roe deer in a temperate forested area. *Chronobiol. Int.* **30,** 772–85 (2013).

15. Bonnot, N. *et al.* Habitat use under predation risk: Hunting, roads and human dwellings influence the spatial behaviour of roe deer. *Eur. J. Wildl. Res.* **59,** 185–193 (2013).
